# Arl13b interferes with α-tubulin acetylation

**DOI:** 10.1186/2046-2530-4-S1-P73

**Published:** 2015-07-13

**Authors:** P Pintado, C Seixas, D C Barral, S S Lopes

**Affiliations:** 1 Cilia Regulation and Disease Lab, CEDOC Chronic Diseases Research Center, NOVA Medical School / Faculdade de Ciências Médicas, Universidade Nova de Lisboa, Campo Dos Mártires Da Pátria, 130, 1169-056 Lisboa, Portugal; 2 Membrane Traffic in Infection & Disease, CEDOC Chronic Diseases Research Center, NOVA Medical School / Faculdade de Ciências Médicas, Universidade Nova de Lisboa, Campo Dos Mártires Da Pátria, 130, 1169-056 Lisboa, Portugal

## Background

The loss of Arl13b has been associated with cilia defects since 2007 [1]. Arl13b is a small G protein that localizes along the ciliary membrane but there is still no current knowledge about Arl13b ciliary role or effectors. Nevertheless, many studies aiming to understand cilia signaling pathways make use of mild Arl13b overexpression fused with GFP as a ciliary marker.

## Objective

Study the impact of overexpressing Arl13b-GFP in ciliary formation and structure.

## Methods

We used the zebrafish Kupffer's vesicle as a dynamic ciliary growth system and performed a seven hour time-course experiment comparing the length of cilia measured by Arl13b-GFP or by acetylated α-tubulin. In order to evaluate the specificity of the alterations in a-tubulin acetylation pattern, we overexpressed different ciliary proteins that were also reported to increase cilia length.

## Results

Arl13b-GFP injection increases cilia length and causes a specific decrease in the α-tubulin acetylation of both motile and primary cilia. We noted that this reduction is more accentuated right before the maximum ciliary length is achieved. Moreover, by blocking deacetylation with tubacin we were able to rescue acetylation levels but cilia length is maintained.

## Conclusions

We concluded that Arl13b overexpression causes a specific and significant reduction in α-tubulin acetylation. We are currently investigating if there is any synergy between the loss of Mec17, the acetylase, and the overexpression of Arl13b. We hypothesize that Arl13b actively blocks α-tubulin acetylation to render the cilium more dynamic and allow it to grow more in the same time window.

## References

[B1] Caspary T Larkins CE Anderson KV The graded response to Sonic Hedgehog depends on cilia architecture Dev Cell 2007 12 5 767 778 10.1016/j.devcel.2007.03.004 17488627

